# Does the Application of Tecar Therapy Affect Temperature and Perfusion of Skin and Muscle Microcirculation? A Pilot Feasibility Study on Healthy Subjects

**DOI:** 10.1089/acm.2019.0165

**Published:** 2020-02-04

**Authors:** Ron Clijsen, Diego Leoni, Alessandro Schneebeli, Corrado Cescon, Emiliano Soldini, Lihui Li, Marco Barbero

**Affiliations:** ^1^ Rehabilitation Research Laboratory (2rLab), Department of Business Economics, Health and Social Care, University of Applied Sciences and Arts of Southern Switzerland, Landquart, Switzerland.; ^2^ Thim Van Der Laan AG, International University of Applied Sciences THIM, Landquart, Switzerland.; ^3^ Faculty of Physical Education and Physical Therapy, Vrije Universiteit Brussel, Brussels, Belgium.; ^4^ Department of Business Economics, Health and Social Care, University of Applied Sciences and Arts of Southern Switzerland, Manno, Switzerland.; ^5^ Department of Innovative Technologies, University of Applied Sciences and Arts of Southern Switzerland, SUPSI, Manno, Switzerland.

**Keywords:** diathermy, physical therapy modality, perfusion imaging, regional blood flow, laser speckle contrast imaging, skin temperature

## Abstract

***Background:*** Tecar therapy (TT) is an endogenous thermotherapy used to generate warming up of superficial and deep tissues. TT capability to affect the blood flow is commonly considered to be the primary mechanism to promote tissue healing processes. Despite some preliminary evidence about its clinical efficacy, knowledge on the physiologic responses induced by TT is lacking.

***Objective:*** The aim of this quantitative randomized pilot study was to determinate if TT, delivered in two modes (resistive and capacitive), affects the perfusion of the skin microcirculation (PSMC) and intramuscular blood flow (IMBF).

***Design:*** A randomized controlled pilot feasibility study.

***Subjects:*** Ten healthy volunteers (*n* = 4 females, *n* = 6 males; mean age 35.9 ± 10.7 years) from a university population were recruited and completed the study.

***Intervention:*** All subjects received three different TT applications (resistive, capacitive, and placebo) for a period of 8 min.

***Outcome measures:*** PSMC, IMBF, and the skin temperature (ST) were measured pre- and post-TT application using power Doppler sonography, laser speckle contrast imaging (LSCI), and infrared thermography.

***Results:*** Compared with placebo application, statistically significant differences in PSMC resulted after both the resistive (*p* = 0.0001) and the capacitive (*p* = 0.0001) TT applications, while only the resistive modality compared with the placebo was capable to induce a significant change of IMBF (*p* = 0.013) and ST (*p* = 0.0001).

***Conclusions:*** The use of power Doppler sonography and LSCI enabled us to evaluate differences in PSMC and IMBF induced by TT application.

## Introduction

The Tecar (capacitive and resistive energetic transfer) is an endogenous thermotherapy that uses electrical currents, induced by a 448 kHz capacitive/resistive monopolar radiofrequency, to generate warming up of deep tissues.^1,2^ Its use in clinical practice has been relatively common for nearly 20 years, but only a few recent studies investigated its clinical efficacy. Most of them reported encouraging results in decreasing pain and improving function in different musculoskeletal clinical conditions as low back pain,^1,3,4^ insertional tendinopathies of the Achilles, the patellar, and the wrist extensor common tendons.^5^ Its capability to affect the blood flow, as a consequence of its thermotherapeutic effect, is commonly considered one way in which TT supports the healing processes of injured/dysfunctional tissues.^6^ Nevertheless, a substantial lack of knowledge exists on the question: does TT affect blood flow in superficial tissue layers?

The Tecar device provides two different treatment modes: capacitive (CAP) and resistive (RES). These modes are normally delivered with different probes (electrodes), made of medical stainless steel. According to Tecar's developers, the two treatment modes induce different tissue responses depending on the resistance of the treated tissue. When the active electrode is provided with an insulating ceramic layer, acting as a dielectric medium, (CAP) the energetic transmission generates only heat in superficial tissue layers, with a selective action on low-impedance (water rich) soft tissues, for example, adipose tissue, muscle, cartilage, and lymphatic system. If the active electrode has no insulating layer, (RES) the radiofrequency energy passes directly through the body in the direction of the inactive electrode, generating heat in the deeper more resistant (low water content) tissue layers, for example, bone, muscular facia, capsules, and tendons. A recent study on healthy volunteers concluded that the delivery of the TT in a mixed mode (described as a “capacitive/resistive”) enhances blood flow volume in muscle tissue.^2^ To the best of knowledge, this pilot project is the first experimental study evaluating the effect of TT (CAP and RES separately) on the perfusion of skin microcirculation (PSMC) and intramuscular blood flow (IMBF) using laser speckle contrast imaging (LSCI) and power Doppler sonography. The aim of this quantitative pilot study is to determine if TT, administered in two modes, affects the IMBF, PSMC, and skin temperature (ST) in healthy subjects. Furthermore, the authors want to estimate variability to determine the sample size for future clinical trials evaluating the physiologic responses of TT.

## Materials and Methods

A sample of 10 healthy subjects (*n* = 4 females, *n* = 6 males; mean age 35.9 ± 10.7 years, mean height 175.7 ± 9.3 cm, mean weight 72.8 ± 12.6 kg) was recruited from a university population. The first 10 volunteers meeting the inclusion criteria were admitted to the study. The inclusion criteria were nonpainful full active range of motion for the right shoulder, elbow, wrist, hand, and cervicothoracic spine. Exclusion criteria were lack of consent in receiving TT, use of pacemaker, epilepsy, angina pectoris, cardiovascular pathologies, pregnancy or breastfeeding, skin lesions, current or recent neck or upper extremity pain (at least 3 consecutive days in the past 6 months), nervous system disorders, diabetes mellitus, thermal sensitivity dysfunction, upper extremities, breast or cervical spine surgery, drug or alcohol abuse, tumors, radiation therapy or chemotherapy in the past year, metallic implants, and internal infection with encapsulated abscess.

All experimental sessions were performed between April 9, 2018 and May 15, 2018, in the Rehabilitation Research Laboratory of the Department of Business Economics, Health and Social Care, University of Applied Sciences and Arts of Southern Switzerland (Manno, Switzerland). Ethical approval was granted by the Ethics Committee of Canton Ticino (2018-00271/CE3327), and the procedures were conducted according to the Declaration of Helsinki. All subjects signed written informed consent before the study. The proposed methodology was developed according to the Consolidated Standards of Reporting Trials statement (2010) containing an extension for a pilot and feasibility trial.^7^

A Tecar device (T-Plus; Wintecare SA, Chiasso, Switzerland) was used to administer the treatment. LSCI (moorFLPI-2; Moor Instruments Ltd., Devon, United Kingdom) and a power Doppler (MyLab Class C; Esaote S.p.a, Genoa, Italy) with a linear probe (LA 533) were used to assess PSMC and IMBF, respectively, according to previous studies investigating similar outcome variables.^2,8–10^

Heart rate (HR), blood pressure (BP), and ST were measured, respectively, with a digital sphygmomanometer (BM 85, Breuer, Ulm, D) and an infrared thermography device (Infrared IR 500-8S, Voltcraft, Hirschau, D). A digital hygrometer and thermometer (Multimeter Voltcraft MT51, Voltcraft, Hirschau, D) were used to measure room temperature and room humidity. A wooden frame for the right upper arm was used to standardize the position during all the experimental procedures.

Three experimental sessions were planned for each participant to test the three different Tecar modes separately: RES, CAP, and placebo (PLAC) separately. To prevent carryover effects, a wash-out period of 1 week between the treatment sessions was considered to be more than sufficient. The order of treatment modality was randomized by asking each participant to choose between three sealed envelopes at the beginning of session I and II. All Tecar applications were performed by the same Tecar-certified physiotherapist.

TT was administered in the RES modality using a round-shaped low-impedance electrode made of medical stainless steel, while the CAP modality TT was delivered using a high-impedance electrode made of medical stainless steel with ceramic coating. In the PLAC modality, TT was delivered by alternating between the high- and low-impedance electrodes with the device switched off. The PLAC application was included to test the potential variation of the blood flow related to the mechanical effect of the probe manipulation and not to the physiologic effect of the TT itself.

**FIG. 1.**
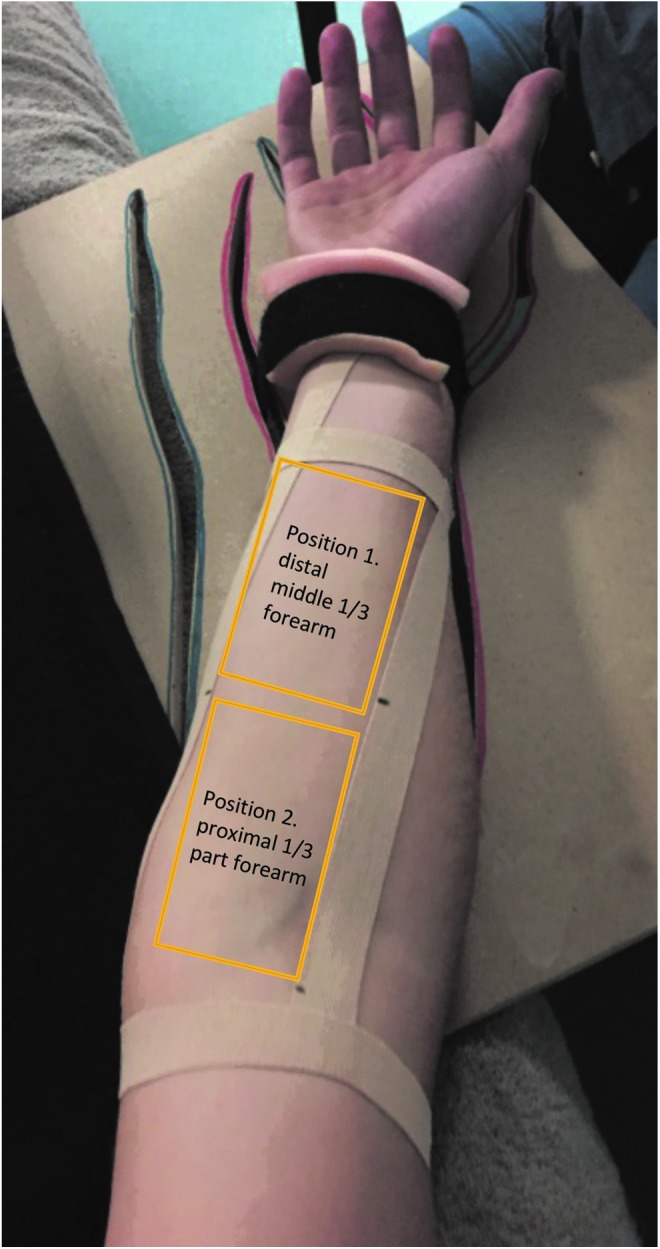
Standardized reference frame for the application of Tecar therapy.

TT was applied to the same region of the right forearm during each of the three sessions. The area was standardized using a reference system based on anatomical landmarks and defined with four strips of tape (Fig. 1), applied during the acclimatization phase at the beginning of each session. The same reference system was used to standardize the position of all the measurement devices. Two pen marks on the lateral strip of tape helped to standardize the position of the power Doppler's probe. In addition, a picture of that frame was taken in session I to have a standardized reference for the following sessions II and III.

The Tecar's plate electrode (inactive electrode), coated with conductive cream, was positioned under the right scapular region before the subjects were placed in a supine position. Following the completion of all premeasurements, a sufficient amount of conductive cream was applied to the skin of the volar forearm to facilitate optimal gliding of the Tecar's probe, and an 8-min TT application was administered. The round-shaped probe was handled with a roto-translatory movement. Each movement cycle lasted 1 sec. A metronome was used to ensure a precise handling of the probe.

The intensity of each mode was set according to a previous pilot testing to ensure the highest possible tolerable treatment intensity. In detail: RES (70%), CAP (40%), and PLAC (0%). In the event that the heat generated by the TT was no longer tolerable, the intensity was decreased as follows: RES (35%) and CAP (20%). After each application, the conductive cream was gently removed from the skin using a cotton towel.

A standard explanation of the experimental procedure was given to the subjects. They were instructed to communicate the heat intensity during the TT application. Subjects were in a supine position with legs straight and both upper arms beside the body. A soft pillow was positioned under the head to avoid discomfort. The right upper arm was positioned at 20° of shoulder abduction, the elbow in full extension, and the forearm was held in full supination by fixing the wrist to the wooden positioning frame with a Velcro strap. Subjects were asked not to move their hand and fingers, to avoid interference with the effect of the Tecar application, respectively, to impair the accuracy of the LSCI and Doppler measurements.

According to similar previous studies,^2,10^ before the measurements all subjects respected a 20-min acclimatization period before any experimental procedure was started. During this time, the subjects rested for 20 min in a supine position in a quiet and darkened room to guarantee stable HR and BP values and the stability of these parameters for the duration of the measurements.

All measurements were performed under standardized laboratory conditions. To compare the effect of TT, pre- and postmeasurements of PSMC, IMBF, HR, BP, and ST were conducted immediately after the TT application (post 1), at time interval 2 min (post 2), and after 10 min (post 3).

The power Doppler images were assessed in two different positions on the volar forearm and arm: Position 1: between the distal and the middle 1/3 of the volar forearm and Position 2: proximal third part of the volar forearm (Fig. 1). In both positions, the probe of the power Doppler was positioned transversally to the longitudinal axis of the forearm, to detect the blood vessels of the transverse section of the wrist flexor muscles at a maximum depth of 3–4 cm.

Two B-mode images, one in each position, were taken before assessing the power Doppler clips. Six power Doppler clips (5 sec long), three in each position, were taken before and immediately after the Tecar application (post 1). The same procedure was repeated at time interval post 2 (2 min) and post 3 (10 min) post-TT application.

The LDCI measurements were performed with a wavelength of 750 nm in a supine position. One day before the measurements, the LDCI system was successfully calibrated. To minimize the risk of confounding factors, daylight and other sources of light were diminished as well as movements of the system during the measurements. The subjects were instructed to breathe normally and not to talk or move during the measurements. The laser aiming function of the device was used to obtain the optimal distance between the measured skin area and the LSCI-system. An *a priori* specified region of interest (ROI) was marked on the volar forearm to obtain standardized values. High-resolution LSCI images (752 × 580 pixels), at a frame rate of 25 Hz (1 sec/frame with an interval of 5 sec), were recorded. The LSCI device uses arbitrary units that reflect the mean flux of an area of interest. The flux is related to the concentration of moving red blood cells in the tissue sample volume, where the level of flux is scaled from blue (low perfusion) to red (high perfusion). LSCI is a relatively new valid method to assess blood flow in the microcirculation of the skin. Compared with laser Doppler flowmetry and laser Doppler imaging, LSCI has some potential advantages. The macroscopic noncontact measurements, with a high spatial and temporal resolution, allow large full-field imaging of the skin microcirculation in real time due to a faster signaling processing.^11,12^ Especially in experimental settings where surface contact is undesirable and microcirculatory perfusion measurements of large skin areas are required, the reproducibility of cutaneous blood flow seems to be superior when measured with LSCI.^13,14^

The Shapiro–Wilks test revealed a non-normal distribution, and thus, data were described using median and interquartile range. The Friedman test was used to analyze the data for statistically significant median differences between pre- and postmeasurements within the three Tecar modes. Bonferroni correction for multiple comparisons was applied. Averages, medians, and standard deviations of all parameters were calculated to describe pre- and postmeasurements as well as their difference.

All statistical analyses were executed using the SPSS statistical package software (IBM SPSS Statistics version 24; SPSS, Inc., Chicago, IL). The significance level was set to *p* < 0.05.

Clips from the power Doppler were analyzed to extract three parameters: (1) area of blood vessels, (2) number of identified vessels, and (3) median power intensity of blood flow. The procedure for the extraction of these three parameters is described in the following paragraphs. The clips from power Doppler were recorded at 11 fps and were 10 sec long, to include at least three systolic and three diastolic movements. Each frame consisted of an image with two superimposed layers: b-mode and blue shade power Doppler image. The pixels with blue shades referred to an arbitrary scale ranging from 0 to 255 according to the ratio between blue and red color intensity in the RGB color code. An arbitrary threshold was set to 100 to avoid measuring background noise that was always present in the images.

The number of pixels with a blue color intensity above 100 was computed for each frame. Since the pixel area curve was increasing during systole and decreasing during diastole, the authors used that curve to identify the systolic and diastolic cycles. The frame indexes corresponding to three systolic and three diastolic movements were identified from the curve of the blood vessels' area and those frames were used to extract the variables of interest. The area of blood vessels was evaluated as described above. To estimate the number of vessels from the images, the individual areas with at least 10 pixels not connected to each other were counted. A threshold of 10 pixels was set to avoid the measurement of background noise.

**FIG. 2.**
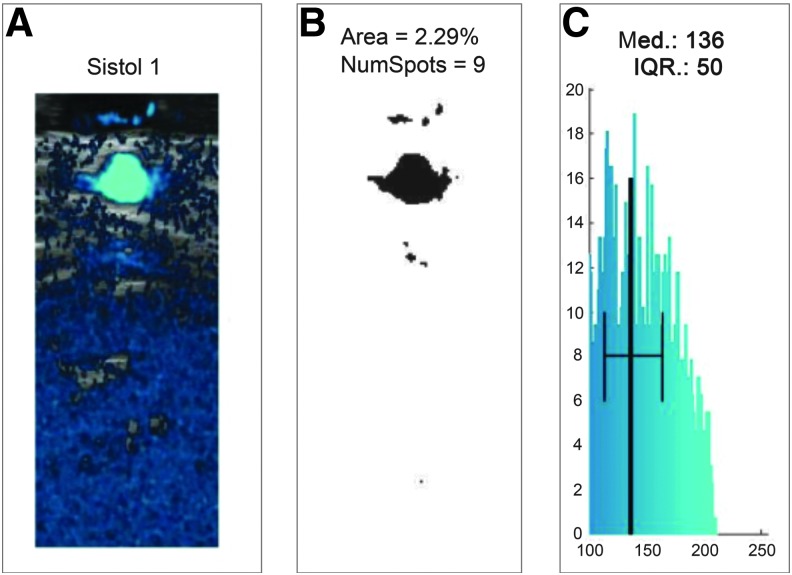
**(A)** Example of a power Doppler image with background noise. **(B)** Example of a filtered power Doppler image for the calculation of the blood flow area. **(C)** Histogram of blue color intensity computed in the extracted area.

An example of this procedure of noise filtering and calculating the area is presented in Figure 2, where Figure 2A represents the power Doppler acquisition with background noise and Figure 2B the filtered image for the calculation of the blood flow area. The final parameter evaluated was the median power intensity of blood flow. This value was derived from the histogram of blue color intensity computed in the area extracted (Fig. 2C) ranging from 0 to 255 on an arbitrary color scale.

**FIG. 3.**
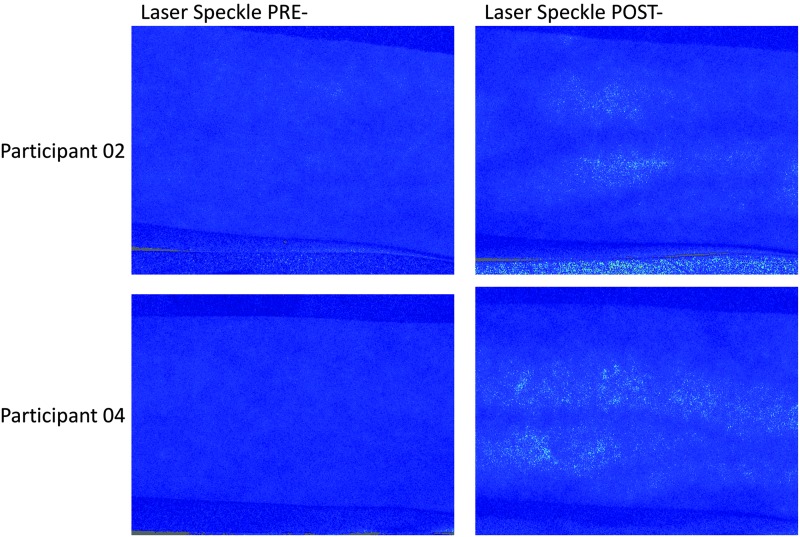
Laser speckle contrast images before and after resistive Tecar application. The *brighter dots* in the postimages represent an increase of skin perfusion.

Due to fluctuations in the values between the two BP phases, it was decided to analyze only those images taken during the diastole. Using the statistical function from the moorFLPI Review V5.0 analysis software, the mean perfusion values of the ROI from the five recorded frames for every measurement interval (pre- and post-TT) were calculated (Fig. 3).

For the following variables, PSMC, IMBF (proximal and distal), HR, and mean arterial pressure (MAP), the median difference between pre- and postintervention measurements was calculated and reported as a percentage. The MAP was calculated from systolic BP (SBP) and diastolic BP (DBP), using the formula as follows: MAP = (SBP +2 × DBP)/3.

## Results

All 10 volunteers successfully completed the three experimental sessions. The experimental procedure and the subjects' position were described as tolerable and comfortable, and none of them reported any adverse effects. Room temperature and room humidity values (mean 24.1°C ± 2.1 and 36.9°C° ± 2.0, respectively) measured immediately before and after the Tecar application were stable enough to assume that any potential difference between pre- and postmeasurements was not influenced by them. Statistically significant differences (*p* < 0.05) of the median difference between each pre- and postintervention measurement were found for PSMC (PLAC—CAP, and CAP—RES), IMBF distal (PLAC-post 1—RES-post 3, CAP-post 2—RES-post 3), and ST (PLAC—RES). For both the control variables, HR and MAP, no statistically significant differences were found (Table 1). Pre- and postabsolute values are reported in Supplementary Table S1.

**Table 1.** Pre- and Postdifferences of the Selected Outcome Measurements

Outcomes	Placebo	Capacitive	Resistive
IMBF, proximal (%)			
Pre- and post 1-difference	0.01 (0.7)^a^	0.36 (4.4)	0.53 (1.04)
Pre- and post 2-difference	0.17 (1.6)	0.79 (3.3)^b^	2.2 (1.95)
Pre- and post 3-difference	0.05 (1.1)	−0.09 (1.9)	2.06 (3.3)^a,b^
IMBF, distal (%)			
Pre- and post 1-difference	−0.2 (1.1)	1.4 (4.06)	1.4 (3.02)
Pre- and post 2-difference	0.5 (1.9)	0.5 (5.2)	1.12 (3.95)
Pre- and post 3-difference	0.1 (2.1)	0.65 (1.13)	1.5 (3.69)
Skin perfusion (%) PSMC			
Pre- and postdifference	−24.8 (16.8)^c,d^	−3.97 (22.01)^c^	23.1 (56.4)^d^
Heart rate (bpm)			
Pre- and postdifference	−1 (7)	1.5 (9)	0.5 (10)
Mean arterial pressure (mmHg)			
Pre- and postdifference	−4.2 (6)	−2.8 (6.3)	0.65 (5)
Skin temperature (°)			
Pre- and postdifference	−2.3 (1.5)^e^	0.9 (1.3)	2.8 (2)^e^

All values are reported as median and IQR.

a–e Indicate significant differences (*p* < 0.05) between groups.

IMBF, intramuscular blood flow; IQR, interquartile range; PSMC, perfusion of the skin microcirculation.

## Discussion

Although TT has been widely used in physical therapy practise as a physical therapy agent for almost 20 years, there are only a few studies that have investigated its clinical efficacy.^1,3^ There seems to be a substantial lack of knowledge on the physiologic responses induced by TT application. A better understanding of the physiologic effects of TT would help in defining the mechanisms underlying its clinical efficacy. From the perspective of evidence-based practice, it would be ideal to base the optimal dosage and choice of mode of a TT application on sound physiologic knowledge. This pilot study on healthy volunteers was, to knowledge, the first attempt to estimate the effect of the TT on blood flow when delivered in two distinct modes: RES and CAP. Ten healthy volunteers were recruited and completed the study. All subjects received three different TT applications (RES, CAP, and PLAC) for a period of 8 min, with an interval of 1 week between applications. PSMC, IMBF, and the ST were measured pre- and postintervention. To minimize the risk of bias, standardization of the procedures (definition of the treatment area, Tecar delivery modes, and measurement acquisition) was planned carefully. Compared with the PLAC application, both the RES and CAP modes were found to be effective in inducing a change in the PSMC. Unexpectedly, the CAP mode induced a slight decrease, while the RES mode, as expected, induced a moderate increase in the PSMC. Regarding the change in IMBF, a significant increase was found for the RES mode at the proximal third of the forearm. In comparison with placebo, no significant changes in IMBF were found for either the CAP or for the RES mode in the measurement at the distal middle third of the forearm.

It should be noted that the significant difference in IMBF, for the RES mode, was only found proximally (position 2: at the proximal third of the forearm), and 10 min after the TT application. A possible explanation for this could be the larger cross-sectional area and consequently vascularization of the forearm (at the site of the proximal measurements) in comparison with the position 1 region (where distal measurements were taken). The onset of change in IMBF needs further investigation. Changes in PSMC and IMBF seem not to be related to any systemic cardiovascular responses, since both HR and MAP did not vary between pre- and postmeasurements, suggesting that TT affects blood flow only at a local level. Interestingly, the difference between pre- and postvalues of ST was only significant when comparing the RES mode with the PLAC mode, with an increase and a decrease of ∼2°C, respectively. These results are in line with Kumaran and Watson^2^ stating that TT has the capability to induce a response in the deep tissue without an excessive increase of ST, a remarkable feature for any intervention with a thermotherapeutic effect.^2^ A limitation that might have influenced the decrease in ST between the RES mode and PLAC mode is the use of a “cold” (room temperature) steel electrode and conductive paste during the application in the PLAC mode. This might have influenced the ST and could have caused a vasoconstrictive effect on the PSMC. The use of thermotherapy is still quite common among physiotherapists, and also of interest to researchers to evaluate the forms of thermotherapy in the management of a variety of musculoskeletal disorders.^15–18^

The ability to induce a thermotherapeutic effect in the deep target tissue (deep muscle layers, joints, and tendons) without generating an excessive increase of the superficial (skin) temperature makes TT highly tolerable for the patients and suitable for the treatment of a variety of musculoskeletal disorders. Therefore, TT applications could be a treatment option especially in conditions where a dysfunction in blood flow plays an important role in generation and persistence of pain and dysfunction (i.e., osteoarthrosis, tendinopathies, and myofascial pain syndrome).^1,3–5,15–22^ Although tendons are poorly vascularized and tendon nutrition is more reliant on synovial fluid diffusion than vascular perfusion, the role of changed vascularization during healing of tendon healing is still not clear.^23^ It remains questionable if TT can affect blood flow in other anatomical structures such as tendons and joint capsules. A previous published study of Kumaran and Watson^2^ suggested that TT application can affect the volume and velocity of IMBF. Compared with this research, some methodological differences can be pointed out. The main difference was in the way in which TT was applied. Kumaran and Watson^2^ delivered TT combining 10 min of the RES mode immediately followed by 5 min in the CAP mode, for a total treatment period of 15 min,^2^ although this option is more similar to commonly used clinical treatment sessions. Combining the two modes does not allow researchers to discriminate if the changes in blood flow differ or depend on the mode delivered. Furthermore, the intensity of the treatment was not standardized for all subjects but was set depending on the perceived local thermal sensation that can be potentially different for every participant. Defining the treatment intensity this way implies a risk of increasing the heterogeneity of the blood flow variations, as it is likely that increasing the treatment intensity might also affect the response size.

Concerning the use of pulsed wave Doppler for blood flow measurement, some differences between the two studies exist, particularly concerning the probe placement. In each participant, Kumaran and Watson used the most prominently identifiable pulsatile (arterial) flow, then following this, the probe was placed parallel to the longitudinal axis of that arteria, to detect its longitudinal section.^2^ Conversely, in the present study, the probe was placed perpendicularly to the forearm to detect the transverse section of regional vessels. This method allowed us to detect blood flow changes occurring not only in one large vessel but also in the smaller ones. This could be considered to a more accurate and suitable method to detect changes in blood flow within the muscle tissue.^8,24^

Despite all the methodological differences, it is encouraging that both the present pilot study and the study of Kumaran and Watson^2^ reported similar results concerning the potency of the TT to affect IMBF. Although the operator's efforts to utilize the same amount of pressure when placing the Tecar's probe on the treated area, no precise measurement of the pressure applied on the skin was carried out during the TT application.

This limitation might have partially affected the variations in blood flow during the three application modes. Other limitations could be the chosen measurement intervals after the TT intervention and the fact that the authors could only measure PSMC before and directly after TT application. The present study found a significant increase of IMBF for the RES mode, which provides support for mechanism of action. It should be stated that this does not necessarily mean that this difference is clinically important or meaningful to patients. Future studies investigating the effect of TT on IMBF and PSMC postintervention measurement should be evaluated in 1-min intervals to get more precise information on the onset of microcirculation blood flow changes.

## Conclusions

In conclusion, these results indicate that TT applications in RES and CAP mode significantly effect PSMC in comparison with a TT PLAC application. A significant change in IMBF and ST was found only for the TT RES mode in comparison with the PLAC application. As the changes in PSMC and IMBF were not related to any systemic cardiovascular responses, TT seems to affect blood flow only at a local level. The applied method using LSCI and the power Doppler technique enabled us to estimate the change in PSMC and IMBF. To improve the clinical efficacy of TT, future studies should focus on physiologic differences between the different TT treatment modes.

## Supplementary Material

Supplemental data
