# Supplementary material for: Does the Application of Tecar Therapy Affect Temperature and Perfusion of Skin and Muscle Microcirculation? A Pilot Feasibility Study on Healthy Subjects
Source: J Altern Complement Med. 2020 Feb 4;26(2):147–53. doi: 10.1089/acm.2019.0165 (PMC7044785; doi:10.1089/acm.2019.0165)
Supplement: Supplemental data [file Supp_Table1.pdf]

Supplementary Data

Supplementary Table S1. Pre- and Postabsolute Values of the Selected Outcome Measurements

Outcomes	*Placebo*	*Capacitive*	*Resistive*
IMBF, distal (%)			
Preintervention values	0.80 (0.73)	2.88 (2.59)	2.83 (1.44)
Post 1 values	0.72 (0.72)	2.80 (2.33)	4.03 (2.30)
Post 2 values	0.68 (1.85)	3.15 (2.07)	4.38 (2.39)
Post 3 values	0.92 (1.37)	3.07 (1.66)	5.44 (2.74)
IMBF, proximal (%)			
Preintervention values	3.01 (6.48)	6.34 (4.06)	6.62 (8.67)
Post 1 values	2.60 (7.12)	7.29 (10.3)	8.97 (8.23)
Post 2 values	2.91 (9.14)	9.00 (8.39)	10.37 (8.43)
Post 3 values	2.95 (7.32)	8.30 (8.64)	9.57 (7.76)
Skin perfusion (arbitrary units) PSMC			
Preintervention values	47.29 (13.99)	47.88 (10.22)	49.10 (9.51)
Postvalues	36.69 (10.14)	48.22 (10.38)	66.38 (22.14)
Heart rate (bpm)			
Preintervention values	63 (13)	61 (9)	61 (11)
Postvalues	65 (9)	63 (8)	61 (4)
Mean arterial pressure (mmHg)			
Preintervention values	95 (9.8)	87.8 (14.1)	87.5 (15)
Postvalues	91 (11.9)	88.5 (9.92)	89.5 (14.67)
Skin temperature (°)			
Preintervention values	33.3 (0.9)	33.2 (1.1)	33.3 (1.2)
Postvalues	30.5 (1.3)	34.6 (1.2)	35.9 (2.3)

All values are reported as median and IQR.
IMBF, intramuscular blood flow; IQR, interquartile range; PSMC, perfusion of the skin microcirculation.
